# The Effect of a Single Freeze–Thaw Cycle on Matrix Metalloproteinases in Different Human Platelet-Rich Plasma Formulations

**DOI:** 10.3390/biomedicines9101403

**Published:** 2021-10-06

**Authors:** Kaitlyn E. Whitney, Grant J. Dornan, Jillian King, Jorge Chahla, Thos A. Evans, Marc J. Philippon, Robert F. LaPrade, Johnny Huard

**Affiliations:** 1 Steadman Philippon Research Institute, Vail, CO 81657, USA; kwhitney@sprivail.org (K.E.W.); gdornan@sprivail.org (G.J.D.); jilliantking@gmail.com (J.K.); 2 Rush University Medical Center, Midwest Orthopaedics at Rush, Chicago, IL 60612, USA; jchahla@msn.com; 3 The Steadman Clinic, Vail, CO 81657, USA; tevans@thesteadmanclinic.com (T.A.E.); drphilippon@sprivail.org (M.J.P.); 4 Twin Cities Orthopaedics, Edina, MN 55435, USA; laprademdphd@gmail.com

**Keywords:** platelet-rich plasma (PRP), leukocyte-poor PRP (LP-PRP), leukocyte-rich PRP (LR-PRP), matrix metalloproteinases (MMPs), freeze–thaw

## Abstract

Storing platelet-rich plasma (PRP) for future use is a compelling approach, presuming the retention of biological properties is maintained. However, certain factors in PRP preparations have deleterious effects for the treatment of certain musculoskeletal conditions. The purpose of this study was to measure and compare matrix metalloproteinase protein (MMP) concentrations between fresh and freeze-thawed leukocyte-rich PRP (LR-PRP) inactivated (LR-I) and activated (LR-A) preparations, and leukocyte-poor PRP (LP-PRP) inactivated (LP-I) and activated (LP-A) preparations. A volume of 60 mL of whole blood was drawn from 19 healthy donors. LP-I and LR-I samples were processed using a manual extraction and centrifugation methodology. LP-A and LR-A products were activated with 10% CaCl_2_ and recombinant thrombin. Blood fractions were either immediately assayed and analyzed or stored at −80 °C for 24, 72 and 160 h. Multiplex immunoassay was used to measure MMP-1, MMP-2, MMP-3, MMP-9, MMP-10, and MMP-12. MMP-1 concentrations increased in LR-A (*p* < 0.05) and MMP-9 significantly increased in LR-I (*p* < 0.05), while MMP-2 significantly decreased in LR-I (*p* < 0.05) and MMP-3 concentrations significantly decreased in LR-A (*p* < 0.05). MMP-12 concentrations also significantly decreased in LR-I (*p* < 0.05) from baseline concentrations. There were no significant differences between LP-A and LP-I preparations and MMP concentrations. MMP-10 concentrations in all PRP samples compared to each freezing time point were also not significantly different. MMPs regulate components of the extracellular matrix (ECM) in the remodeling phase of musculoskeletal injury. In this study, we observed a significant increase and decrease in MMP concentrations in response to a single freeze–thaw cycle in inactivated PRP and activated PRP preparations. This evidence contributes to the growing body of literature on the optimization of PRP preparation and storage strategies prior to delivery. Our findings suggest that specific PRP preparations after a single freeze–thaw may be more advantageous for certain musculoskeletal applications based on the presence of MMP concentrations.

## 1. Introduction

Musculoskeletal injury is a leading cause of long-term joint dysfunction and disability [1]. Since these injuries often affect young, active individuals, the long-term impact of early-onset osteoarthritis (OA) is of great concern [2]. The ability to prevent or delay post-traumatic OA will help maintain the musculoskeletal health of injured individuals, significantly reduce overall disability claims and dysfunction, and reduce subsequent conditions and co-morbidities [3,4,5]. The use of biological therapeutics for musculoskeletal care is increasing as the molecular and biological understanding of their applications for different orthopedic pathologies are elucidated [6,7,8,9,10]. With the growing demand for alternative orthopedic and sport medicine treatments to accelerate return to activity, platelet-rich plasma (PRP) has emerged as a promising orthobiologic agent for various musculoskeletal applications [11,12,13,14,15]. While there are several initiatives that have committed to improving the clinical utility and evaluation of PRP [16,17,18], the quality and safety of delivering platelet-derived growth factors and other molecular signals to enhance healing have been well documented [8,14]. The clinical utility of different PRP formulations, as it contains components that can alleviate inflammation and degenerative processes [13,19,20], the time to recovery for affected individuals from joint damage may be substantially reduced if immediate PRP intervention is introduced at the time of injury. It is important to note that even though there is evidence suggesting that PRP can enhance musculoskeletal healing, there is still uncertainty around the biological mechanisms that are responsible for stimulating musculoskeletal tissue regeneration and therapeutic responses. 

Platelets have a crucial homeostatic function in the presence of injured tissue by binding to activating receptors that causes downstream signaling to enzyme cyclooxygenase-1 (COX-1) that synthesizes with thromboxane A2 (TXA) to further activate nearby platelets [21]. A fibrin clot develops as platelets continue to activate, secreting factors from the platelet granule compartments up to 24 h after activation [22]. Platelets undergo degranulation after endogenous (calcium chloride, chitosan) or exogenous activation; thereby, releasing different growth factors and other active molecules (chemokines, extracellular matrix, proteins, nucleotides) that have significant roles in the musculoskeletal healing process [12]. Catabolic factors, such as matrix metalloproteinases (MMPs), are one of many factors that are secreted by platelet granules and have highly dynamic roles in musculoskeletal tissue development, repair and degradation (Table 1) [23,24]. In fact, elevated levels of MMPs have been linked to numerous physiologic and pathologic conditions and are predictors of poor tissue healing outcomes [25,26,27]. Recent studies have shown that modulating cytokines, chemokines and growth factors (e.g., vascular endothelial growth factor [VEGF] and transforming growth factor beta-1 [TGF-B1]) found in PRP can improve cartilage and skeletal muscle healing [28,29,30,31,32]. A single freeze–thaw cycle may be a potential strategy to reduce or remove catabolic factors, such as MMPs, that adheres to the FDA’s Human Cells, Tissues, and Cellular and Tissue Based Products (HCT/P’s) provisions [33,34]. 

Thus, effective storage strategies to preserve the biological components of PRP are of the outmost importance. Currently, single extraction procedures for multiple applications or emergency care utilization is the gold standard [51,52]. In this regard, it has been reported that platelets stored for 21 days can retain their activity, but are at a higher risk of bacterial proliferation [53]. Although, cryopreservation methods can potentially reduce the bacterial rise [54], irreversible platelet activation induction can be triggered, shortening the life span of the various proteins and bioactive factors [52]. The paucity of studies demonstrating the short-term freezing effects on the viability and activity of biological constituents in PRP formulations may be in part due to the variety of factors that could influence the stability of the sample when attempting to preserve PRP, ranging from temperature, freezing techniques, and timing, to the inclusion of cryoprotective agents to stabilize and preserve cells and proteins [55,56,57]. Moreover, the lack of universal biobanking guidelines and reporting requirements for storing biospecimens, including PRP, has made it challenging to establish and compare effective storage strategies. Nonetheless, two common storage strategies are generally accepted for complex biospecimens, including freezer storage (−20, −30, −80 °C) and liquid nitrogen storage [58,59]. For long-term storage, ultra-low temperatures (−80 to −196 °C) and liquid nitrogen have been found to not adversely affect sample quality and are primarily used to store tissues that contain cellular and protein material [58,60,61]. To this end, identifying an adequate and practical storage strategy to preserve or reduce biological constituents in PRP in a minimally-manipulative fashion is necessary prior to establishing PRP banking guidelines. In the present study, 4 different PRP preparations were quantitatively measured at 3 different times points over a 7 day period using a single cryogenic storing temperature (−80 °C) using a slow-freeze strategy to address the resulting freezing and freeze–thaw effects. The purpose of this study was to measure and compare MMPs in fresh and freeze-thawed whole-blood, leukocyte-rich PRP (LR-PRP) inactivated (LR-I) and activated (LR-A), leukocyte-poor PRP (LP-PRP) inactivated (LP-I) and activated (LP-A). We hypothesized that short-term freezing (−80 °C) would affect MMP concentrations in whole-blood, inactivated PRP and activated PRP preparations.

## 2. Methods

### 2.1. Subject Enrollment and Demographics

**Figure 1:**
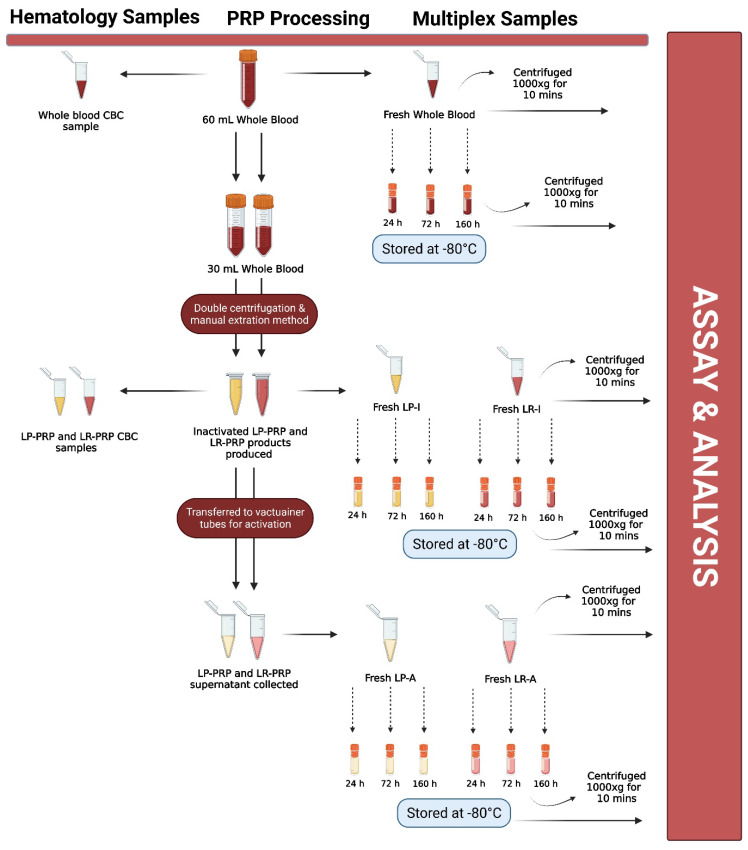
Flow diagram describing each processing and testing step. Leukocyte-rich platelet-rich plasma (LR-PRP); leukocyte-poor platelet-rich plasma (LP-PRP); inactivated leukocyte-poor PRP (LP-I); inactivated leukocyte-rich PRP (LR-I); activated leukocyte-rich PRP (LR-A); activated leukocyte-poor PRP (LP-A); hours (h); minutes (mins); complete blood count (CBC). Figure created with BioRender.com.

After Institutional Review Board (IRB) approval (Vail Health Ethics Committee; protocol 2017-36), twenty-four healthy donors between 18 and 70 years of age were enrolled in this study. Informed consent was obtained from all subjects involved in the study. Demographics were collected from each study participant, including sex, age, height, weight and body mass index (BMI). The minimum information for studies evaluating biologics in orthopedics (MIBO) and STROBE guidelines were adhered to in this study. Subjects were excluded from this study if they were actively taking prescribed medications or non-steroidal anti-inflammatory drugs (NSAIDs) or had a present or previous history of blood immunosuppressive disorders, cancer, osteonecrosis, rheumatoid arthritis or avascular necrosis. Subjects were prospectively enrolled over a 6 week period. A flow diagram is shown in Figure 1 that describes each of the processing steps below. 

### 2.2. Whole-Blood Collection

Subjects underwent a standard venipuncture procedure to draw 60 mL of peripheral blood at the time of enrollment. Briefly, a certified phlebotomist cleansed the skin with an alcohol swab and peripheral blood from the antecubital (AC) fossa area was drawn into a syringe prefilled with 10 mL of anticoagulant citrate dextrose formula-A (ACD-A) (Fenwal Laboratories, Lake Zurich, IL, USA). The syringe was capped and taken to a clinical laboratory for hematology analysis and PRP processing.

### 2.3. Whole-Blood Hematology Analysis

All whole-blood samples were processed using a centrifugation and manual extraction methodology. Under a biosafety cabinet, the 60 mL sample was distributed into two 30 mL increments in separate 50 mL conical tubes (FalconTM Conical Centrifuge Tube). An 800 µL sample of whole blood was pipetted into a microcentrifuge tube (1.5 mL, Seal-Rite^®^, Sterile Microcentrifuge Tube) to collect a complete blood count (CBC) using a hematology analyzer (CellDyn Ruby^®^, Abbott Diagnostic Division, Abbott Park, IL, USA). The hematology analyzer measures platelets, leukocytes and five differentials including neutrophils, lymphocytes, monocytes, eosinophils and basophils in the ×10^3^/microliter (µL), and erythrocytes in ×10^6^/µL. Approximately 400 µL of whole blood was transferred to microcentrifuge tubes labeled with fresh and frozen time points for multiplex immunoassay and analysis. The fresh whole blood was placed on a rocker at room temperature until the assay was prepared, while the labeled frozen samples were stored at −80 °C for 24, 72 and 160 h. 

### 2.4. Inactivated and Activated Leukocyte-Poor PRP Processing Technique

Our initial optimization procedures demonstrated that higher centrifugation speeds and a longer spin duration caused the platelets to drop into the mononuclear layer and erythrocyte layer when processing a LP-PRP product. Therefore, our centrifugation parameters include a centrifugation step at 500× *g* for 10 min with gradual deceleration (no break) to allow the platelets to remain within the plasma layer, as previously described [62]. Briefly, the 60 mL of whole blood was separated into two 30 mL increments in 50 mL conical tubes. The 30 mL whole-blood sample was then centrifuged at 500× *g* for 10 min using a SorvallTM ST 8 benchtop centrifuge (ThermoFisher Scientific, Waltham, MA, USA). After the completion of the first centrifugation step, the conical tubes were taken under a biosafety cabinet and a pipette (FalconTM, Pipet Controller) was used to manually extract the excess plasma layer lying just above the leukocyte “buffy coat” layer. The plasma was transferred to a 50 mL conical tube and a second centrifugation step at 3000× *g* for 6 min was performed. After the completion of the second centrifugation, the excess plasma and leukocyte “buffy coat” layers were extracted and discarded. The cell pellet was resuspended in plasma, resulting in 3 mL of LP-I. Next, 800 µL of LP-PRP was transferred to a microcentrifuge tube for hematology analysis. Lastly, 400 µL of LP-I was transferred to microcentrifuge tubes labeled with fresh and frozen time points for multiplex immunoassay and analysis. The remaining LP-I was transferred to a 10 mL BD Vacutainer (BD, Franklin Lakes, NJ, USA) and 200 µL of 10% CaCl_2_ and 20 µL of recombinant thrombin were added to the vacutainers. The BD Vacutainer was inverted 5 times and left upright for 30 min at room temperature to allow the platelets to aggregate and the clot to form. After 30 min, approximately 200 µL of the supernatant was extracted and transferred from the BD Vacutainer to microcentrifuge tubes labeled with fresh and frozen time points. The fresh LP-I and LP-A samples were placed on a rocker at room temperature until the assay was prepared, while the labeled frozen samples were stored at −80 °C for 24, 72 and 160 h.

### 2.5. Inactivated and Activated Leukocyte-Rich PRP Processing Technique

An initial centrifugation at 1500× *g* for 10 min with gradual deceleration (no break) to allow the platelets to drop to the monolayer in which both platelets and leukocyte cells can be extracted and isolated for further processing was performed following a previously described protocol [62,63]. Briefly, the second 30 mL increment of whole blood was centrifuged at 1500× *g* for 10 min. After the initial centrifugation step, the plasma and leukocyte “buffy coat” layer were extracted and transferred to a 50 mL conical tube. The blood fractions then went through a second centrifugation step at 3000× *g* for 6 min followed by final manual extraction of the excess top fraction of plasma. Approximately 3 mL each of the plasma and leukocyte “buffy coat” layers was extracted and transferred to a 50 mL conical tube, resulting in 3 mL of LR-I. Approximately 800 µL of LR-PRP was transferred to a microcentrifuge tube for hematology analysis. Lastly, 400 µL of LR-I was transferred to microcentrifuge tubes labeled with fresh and frozen time points for multiplex immunoassay and analysis. The remaining LR-I was transferred to a 10 mL BD Vacutainer and 200 µL of 10% CaCl_2_ and 20 µL of recombinant thrombin were added to the vacutainers. The BD Vacutainer was inverted 5 times and left upright for 30 min at room temperature to allow the platelets to aggregate and the clot to form. After 30 min, approximately 200 µL of the supernatant was transferred from the BD Vacutainer to microcentrifuge tubes or cryovials labeled with fresh or frozen time points. The LR-I and LR-A samples were placed on a rocker at room temperature. The fresh microcentrifuge tubes were then centrifuged at 1000× *g* for 10 min to remove the cellular debris and collect the pure plasma and supernatant. All samples were thawed within 30 min of reconstituting the multiplex standards. All labeled cryovial tubes followed the freezing and freeze–thaw procedures described below. 

### 2.6. Freezing and Freeze–Thaw Procedures

Each of the labeled cryovials was placed in a Nalgene^®^ Mr. Frosty^®^ (Sigma-Aldrich, St. Louis, MO, USA) to gradually freeze at −80 °C for 24, 72 and 160 h. No cryoprotective additives were added to the microcentrifuge tubes prior to freezing. 

Following a 24, 72 or 160 h freeze at −80 °C, samples were placed on a rocker at room temperature until thawed. The thawing process took approximately 30 min. A water bath was not used in this experiment due to the risk of sample contamination and represents a clinical scenario. The microcentrifuge tubes were then centrifuged at 1000× *g* for 10 min to remove the cellular debris and collect the pure plasma and supernatant. All samples were thawed within 30 min of reconstituting the multiplex standards. 

### 2.7. Multiplex Immunoassay 

The blood fractions were pipetted into microcentrifuge tubes for Luminex^®^ multiplex immunoassays (EMD Millipore Corp, Billerica, MA, USA) that measured the concentrations of MMP isoforms from panels 1 and 2 kits (EMD Millipore Corp, Billerica, MA, USA) were measured. A standard manufacturer’s protocol for the Luminex^®^ 200 (Luminex Corp, Austin, TX, USA) multiplex instrument was utilized as previously published [64]. All reagents were prepared and stored according to manufacturer’s instructions. Briefly, background, standards, and controls were added in duplicate to the appropriate wells with serum matrix solution. The unknown samples were diluted with assay buffer for panel 2 and neatly ran for panel 1, then subsequently added in duplicate along with premixed antibody immobilized magnetic beads. The plate was sealed and covered with foil during incubation with agitation at 600 RPM. Using a handheld magnet, the plate was washed two times using the 1X wash buffer provided. Detection antibodies were added to the plate and incubated at room temperature for 30 min at 600 revolutions per minute (RPM). Streptavidin-phycoerythrin solution was added and incubated at room temperature for 30 min at 600 RPM. Following two plate washes, drive fluid was added to re-suspend the beads at 300 RPM for five minutes. Finally, the plate was analyzed with the Luminex^®^ 200 xPONENT 3.1 system (Luminex Corp, Austin, TX, USA) using the xPonent^®^ software (EMD Millipore Corp, Billerica, MA, USA), which created a standard curve for each respective analyte utilizing a five-parameter logistic curve-fitting method with the median fluorescent intensity. MMP concentrations in the unknown samples were then calculated. 

### 2.8. Statistical Analysis

An a priori power analysis was formulated based on previous pilot data. We hypothesized that blood-cellularity and protein concentrations in LR-PRP and LP-PRP would statistically differ among the fresh and the three freeze–thaw time points. Assuming analysis of variance (ANOVA), we calculated that 72 PRP samples and 18 donors would be necessary to achieve at least 80% statistical power to test the overall null hypothesis of no group differences (α = 0.05, β = 0.81). Assuming biological variability, we expected that 20 healthy donors and 80 PRP samples (10 per PRP type) would be a sufficient sample size to determine significant differences between the biological composition of PRP preparations. 

The Skillings–Mack test was used to test the overall null hypothesis of equivalency among fresh and the three freeze–thaw preparations within the whole blood and each PRP preparation separately. This method is analogous to a one-way repeated-measures analysis of variance model, and advisable in cases where data may be non-normally distributed, may exhibit extreme values or outliers, or for data that contain missing values [65]. Pairwise comparisons were performed when there was statistical significance using the dependent-samples Wilcoxon signed rank tests. In this case, Holm’s method was used to maintain a familywise alpha error rate of 0.05 among for the six pairwise comparisons. Spearman’s correlation and Mann–Whitney U tests were used to test for associations between factors and demographic covariates. The statistical software R was used for all analyses [66]. 

## 3. Results

### 3.1. Subject Demographics and Complete Blood Count Results

A total of 24 subjects were prospectively enrolled in this study. Five subjects were withdrawn from this study. Two subjects failed the eligibility criteria after screening and three subjects had blood samples that were not viable for assay and analysis and declined a repeat blood draw. Data generated from nineteen healthy donors were used in the final analysis of this study. Subject demographics are reported in Table 2, total white blood cell (WBC), red blood cell (RBC) and platelet (PLT) counts are reported in Table 3, and leukocyte differential counts are reported in Table 4. The effect of freezing on platelet concentrations has been well described [67,68], thus a CBC was only collected on fresh (baseline) whole-blood, LP-I and LR-I samples. In fresh whole blood, there was a positive correlation between MMP-3 and age (rho = 0.73, *p* < 0.001) and a negative correlation between MMP-10 and age (rho= −0.48, *p* < 0.05). There were no significant correlations between age, gender or BMI and fresh (baseline) MMP concentrations. 

### 3.2. Whole-Blood Matrix Metalloproteinase Results 

**Figure 2:**
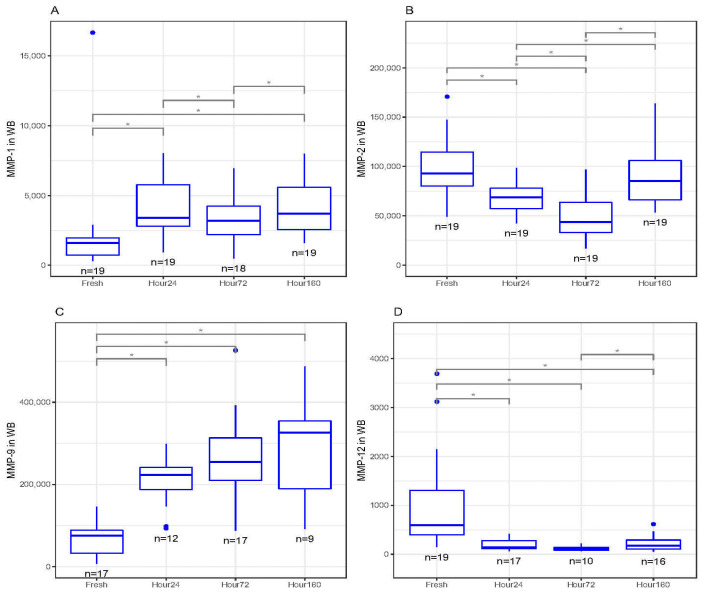
(**A**) Matrix metalloproteinase-1 (MMP-1) concentrations (pg/mL) in whole-blood (WB) freeze–thaw samples significantly increased at 24 and 160 h compared to fresh (baseline) samples (* *p* < 0.05). (**B**) Matrix metalloproteinase-2 (MMP-2) concentrations (pg/mL) in WB freeze–thaw samples significantly decreased at 24 and 72 h compared to fresh (baseline) samples (* *p* < 0.01). (**C**) Matrix metalloproteinase-9 (MMP-9) concentrations (pg/mL) in WB freeze–thaw samples significantly increased at 24, 72 and 160 h compared to fresh (baseline) samples (* *p* < 0.05). (D) Matrix metalloproteinase-12 (MMP-12) concentrations (pg/mL) in WB freeze–thaw samples significantly decreased at 24, 72 and 160 h compared to fresh (baseline) samples (* *p* < 0.05). All boxplots in (**A**–**D**) represent the group median (middle horizontal line), interquartile range (IQR, top and bottom of boxes), range (vertical ‘whiskers’) and outliers that exceed 1.5∗IQR away from the nearest quartile (dots).

In whole blood, MMP-1 concentrations significantly increased between fresh and 24 and 160 h (*p* < 0.05) after freezing (Figure 2A). There were also significant differences in MMP-1 concentrations between 24 and 72 h, as well as 72 and 160 h after freezing (*p* < 0.05, Figure 2A). MMP-2 concentrations significantly decreased between fresh and 24 and 72 h after freezing (*p* < 0.01) but was not statistically different between fresh and 160 h after freezing (Figure 2B). There were also significant differences in MMP-2 concentrations between 24 and 72 h, as well as 72 and 160 h after freezing (*p* < 0.05, Figure 2B). MMP-9 concentrations also significantly increased between fresh and 24, 72 and 160 h after freezing (*p* < 0.05, Figure 2C). Conversely, MMP-12 concentrations significantly decreased between fresh and 24, 72 and 160 h after freezing (*p* < 0.05, Figure 2D). There was also a significant difference in MMP-12 concentrations between 72 and 160 h after freezing (*p* < 0.05, Figure 2D). There were no significant differences between fresh and frozen MMP-3 and MMP-10 concentrations in whole blood. 

### 3.3. Inactivated Leukocyte-Rich PRP Matrix Metalloproteinase Results 

**Figure 3:**
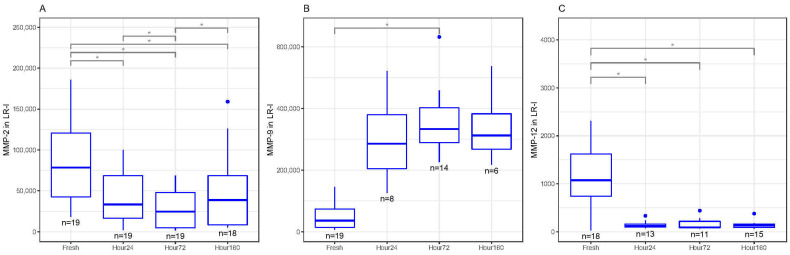
(**A**) Matrix metalloproteinase-2 (MMP-2) concentrations (pg/mL) in inactivated leukocyte-rich platelet-rich plasma (LR-I) freeze–thaw samples significantly decreased at 24, 72 and 160 h compared to fresh samples (* *p* < 0.05). (**B**) Matrix metalloproteinase-9 (MMP-9) concentrations (pg/mL) in inactivated leukocyte-rich platelet-rich plasma (LR-I) freeze–thaw samples had significantly increased at 72 h compared to fresh samples (* *p* < 0.05). (**C**) Matrix metalloproteinase-12 (MMP-12) concentrations (pg/mL) in inactivated LR-I freeze–thaw samples significantly decreased at 24, 72 and 160 h compared to fresh (baseline) samples (* *p* < 0.05). All boxplots in (**A**–**C**) represent the group median (middle horizontal line), interquartile range (IQR, top and bottom of boxes), range (vertical ‘whiskers’) and outliers that exceed 1.5∗IQR away from the nearest quartile (dots).

In LR-I, MMP-2 decreased in concentration significantly between fresh and 24, 72 and 160 h after freezing (*p* < 0.05, Figure 3A). There were also significant differences in MMP-2 concentrations between 24 and 72 h, as well as 72 and 160 h (*p* < 0.05, Figure 3A). MMP-9 increased in concentrations between fresh and 72 h after freezing (*p* < 0.01) but was not statistically significant between fresh and 24 and 160 h after freezing (Figure 3B). MMP-12 significantly decreased in concentrations between fresh and 24, 72 and 160 h after freezing (*p* < 0.05, Figure 3C). There were no significant differences between fresh and frozen MMP-1, MMP-3 and MMP-10 concentrations in LR-I. 

### 3.4. Activated Leukocyte-Rich PRP Matrix Metalloproteinase Results

**Figure 4:**
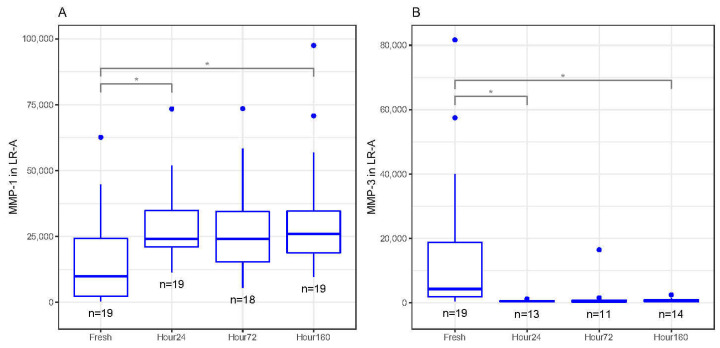
(**A**) Matrix metalloproteinase-1 (MMP-1) concentrations (pg/mL) in activated leukocyte-rich platelet-rich plasma (LR-A) freeze–thaw samples significantly increased at 24 and 160 h compared to fresh (baseline) samples (* *p* < 0.05). (**B**) Matrix metalloproteinase-3 (MMP-3) concentrations (pg/mL) in activated LR-A freeze–thaw samples significantly decreased at 24 and 160 h compared to fresh (baseline) samples (* *p* < 0.05). All boxplots in (**A**–**B**) represent the group median (middle horizontal line), interquartile range (IQR, top and bottom of boxes), range (vertical ‘whiskers’) and outliers that exceed 1.5∗IQR away from the nearest quartile (dots).

In LR-A, MMP-1 significantly increased in concentration between fresh and 24 and 160 h after freezing (*p* < 0.05, Figure 4A). There was an upward trend in MMP-1 concentrations between fresh and 72 h after freezing, but the change in concentration was not significant (Figure 4A). MMP-3 significantly decreased in concentration between fresh and 24 and 160 h after freezing (*p* < 0.05) but was not statistically different between fresh and 72 h (Figure 3B). There were no trends or statistically significant differences between fresh and frozen MMP-2, MMP-9, MMP-10 and MMP-12 concentrations. 

### 3.5. Inactivated and Activated Leukocyte-Poor PRP Matrix Metalloproteinase Results

There were no trends or statistically significant changes in MMP-1, MMP-2, MMP-3, MMP-9, MMP-10 and MMP-12 concentrations between fresh and frozen LP-I and LP-A samples. 

### 3.6. Multiplex Detectability of MMP Isoforms

The MMPs were measured using magnetic bead panels that have been measured in plasma and supernatant from whole-blood and activate/inactivated PRP samples. Certain MMPs exhibited high degrees of skew and missing or nondetectable values. Non-detect results are believed to be due to bead aggregation (clumping), which occurs more often when there are several bead panels present. MMPs for which the detectability rate was at least 80% were included in the final analysis and reporting.

## 4. Discussion

As the burden of musculoskeletal injuries continues to rise [69], the demand for biologic treatment has grown exponentially throughout the years and has enabled basic science and clinical research to bridge many milestones [11]. PRP is a safe biological treatment that can be produced through minimal extraction and preparation methods to deliver a high concentration of platelet-derived growth factors and other bioactive factors [70]. While there have been several clinical research advancements and it is considered a promising alternative to conventional treatments [71], there is little consensus on its standard processing methodology and application [16]. These variations have been shown to affect the PRP product [71], in which the biological composition may be directly related to the processing methodology. Cytokines, chemokines, and growth factors, such as insulin growth factor-1 (IGF-1), transforming growth factor beta-1 (TGF-β1), vascular endothelial growth factor (VEGF), interleukin-1 receptor antagonist (IL-1Ra), platelet-derived growth factor (PDGF) and fibroblast growth factor (FGF), are secreted at varying concentrations over time depending on the PRP formulation [6,62,63,72,73,74,75,76,77]. Certain factors are selectively regulated by granule proteins for either growth factor activation or inhibition [78]. However, endogenous and exogenous mechanisms can induce α-granules activation to secrete high concentrations of anabolic and catabolic growth factors, cytokines and chemokines over a 7 day period [73], the viability of these factors are compromised after 5 days at room-temperature [79]. Chemokines, such as MMPs, are also present in PRP products and regulate components of the extracellular matrix in the inflammatory and remodeling phase of musculoskeletal injury [62]. The possibility of storing PRP for future use is a compelling approach to reduce factors that have degradative roles in musculoskeletal tissue regeneration and repair, presuming that the retention of the biological function is maintained. Currently, little knowledge exists on the effects of a single freeze–thaw cycle and MMP concentrations in different PRP formulations. 

In our study, MMP-1 concentrations increased in whole blood and LR-A and MMP-9 significantly increased in whole blood and LR-I, while MMP-2 significantly decreased in whole blood and LR-I, MMP-3 concentrations significantly decreased in LR-A, and MMP-12 concentrations significantly decreased in whole blood and LR-I from baseline concentrations after a single freeze–thaw cycle. Interestingly, there were no significant differences between LP-A and LP-I MMP concentrations after a single freeze–thaw cycle. To note, freeze–thaw samples of whole-blood MMP-1, MMP-2, and MMP-12 concentrations, as well as LR-I MMP-2 concentrations were found to be significantly different. One explanation to this observation is that the samples were ran on different days and separate multiplex standards which may have caused variation in concentrations between freeze–thaw time points. Although there was variation observed in concentrations between freeze–thaw time points, these concentrations indeed followed increased or decreased concentration patterns when compared to fresh concentrations in whole-blood and LR-I samples. In addition, there were no significant differences between MMP-10 concentrations and all PRP samples after a single freeze–thaw cycle. In fresh whole blood, there was a positive correlation between MMP-3 and age and a negative correlation with MMP-10 and age. 

Several years of research has demonstrated irreversible alterations to platelets following freezing or cooling techniques for storage [57,80,81,82,83,84,85,86]. Despite recent advancements and attempts to extend the shelf-life of platelet concentrates, the 7 days storage strategy remains the gold standard [82,87]. Over time, platelet viability and reactivity diminishes, resulting in variability in platelet aggregation and protein secretion [88,89,90]. Current efforts have mainly focused on retaining anabolic factors following a single freeze–thaw cycle or lyophilization of PRP [51,52,91,92,93,94]. Although a single freeze–thaw cycle is thought to be a more cost-effective alternative for platelet activation, recombinant thrombin and CaCl_2_ are also fast-acting activators that can be used to degranulate platelet-derived proteins in the supernatant of PRP products prior to long-term storage [73,95,96]. However, there is limited evidence demonstrating the detrimental effect of freezing on the biological composition and activity of PRP. Roffi et al. [97] compared freeze-thawed and fresh PRP by measuring a combination of inflammatory, angiogenic and fibrotic/chondrogenic factors, such as IL-1beta, HGF, PDGF AB/BB, TGF-β1, and VEGF, and found significantly lower TGF-β1 and PDGF-AB/BB, while IL-1β and HGF significantly increased after the freeze–thaw process. Similarly, Steller et al. [98] demonstrated increased PDGF AB/BB, TGF-β1, and VEGF concentrations in non-activated PRP samples following a since freeze–thaw cycle. This indicates that PRP samples that are not activated prior to freezing results in rapid platelet degranulation, resulting in an increase in growth factor concentrations in the supernatant or platelet-poor plasma that is subsequently tested for protein levels. More recently, McClain et al. [56] reported significant differences in MMP-9, PDGF-BB, insulin growth factor-1 and TGF-β1 concentrations between fresh and freeze-thawed PRP samples in an equine model. More specifically, they found that MMP-9 concentrations in activated PRP increased between fresh and 1 month following a single freeze–thaw cycles [56]. MMPs are predominately known for maintaining the extracellular matrix and their deleterious effects on musculoskeletal tissues [23,99,100]. The individual roles of MMP isoforms in the setting of soft tissue or joint injury are fairly complex due to their interplay in regulating structural components; however, each MMP isoform has specialized roles in tissue degradation and maintenance, as briefly described in Table 1. In the present study, six MMP isoforms were evaluated to determine the potential denaturation effects of a single freeze–thaw cycle on proteins involved in catabolic processes. We initially hypothesized that the freeze–thaw cycle could serve as a potential minimal manipulation strategy that falls under the HCT/P provisions in order to reduce MMP concentrations in different PRP formulations. Surprisingly, these freezing conditions had individually altered certain MMP isoform concentrations in activated and inactivated PRP preparations. Moreover, MMP-9 concentrations had been found to increase after a single freeze–thaw cycle and our results corroborated McClain et al.’s results in PRP [56]. These changes in MMP concentrations between fresh and short-term freezing time points may be due to structural changes to the leukocytes or platelets that results in complete degranulation or protein denaturation. This is relevant when measuring and comparing the biological profiles, and more specifically catabolic enzymes/proteins that are present in PRP preparations used for musculoskeletal treatment. To this end, further assessment of these catabolic enzymes/proteins present in different PRP formulations is necessary. Additionally, using a single freeze–thaw cycle to reduce concentrations of certain MMP isoforms prior to PRP delivery is an interesting strategy to modify these biological therapies using a minimal manipulation technique. 

In summation, we observed that MMP isoform concentrations increased and decreased in response to a single freeze–thaw cycle in whole-blood, inactivated PRP, and activated PRP formulations. Suggesting that a single freeze–thaw cycle can be a minimal manipulation strategy to reduce concentrations of MMP isoforms. However, this does not represent the functional activity of MMP isoforms in PRP future studies would be necessary to determine the influence of a single freeze–thaw cycle on the function of PRP-derived MMPs in vitro and in vivo. Nonetheless, the development of PRP preservation approaches through minimal manipulation represents an important step in PRP mediated tissue regeneration and repair. In terms of clinical relevance, it has been recently shown that stored PRP can also delay aging through the recovery of stem cell senescence in a preclinical model [101]. Further characterization studies are necessary to investigate the effects of different storage strategies on other growth factors, cytokines and chemokines (i.e., TGF-β, VEGF) that serve roles for soft tissue and cartilage/bone regeneration and repair. 

We recognize that there were some limitations in this study. First, there was variability amongst donors in the PRP cell counts, and the sample size of healthy donors was relatively small. Second, healthy donors are not representative of orthopedic patients that have significantly different systemic profiles; however, it was important to evaluate biological changes after a single-freeze thaw cycle in subjects with normative profiles. Third, the effect of short-term freezing (1 week) on MMP concentrations was evaluated. There may be significant changes in the biological activity of PRP in vivo and MMP concentrations in PRP preparations following long-term freezing that were not significantly altered after short-term freezing. Fourth, although we observed significant differences in MMP concentrations before and after freezing, the effect of freezing on the biological activity of PRP in any given tissue requires further in vitro testing. Fifth, developing approaches through the inclusion of preservative agents to preserve the biological activity of PRP also represents a limitation to our study. There is a lot of work in the area of cryopreservation of blood cells, proteins and progenitor cells that can be explored in the future to potentially preserve the biological activity of PRP. 

## 5. Conclusions

In this study, we found that MMPs significantly increase (MMP-1 and MMP-9) or decrease (MMP-2, MMP-3 and MMP-12) in response to a single freeze–thaw cycle in whole-blood, inactivated PRP, and activated PRP formulations. The possibility of storing PRP, while reducing catabolic factors, for future use is compelling for clinical practice. Although we observed differences in MMP concentration following a single freeze–thaw cycle, the effect of freezing on the biological activity of PRP in a given tissue remains to be determined. Further studies are warranted to determine the influence of long-term freezing on the biological composition and the effects of freezing on the biological activity of PRP. The development of cryopreservation techniques to preserve the biological concentrations in different PRP preparations after freezing is needed for the widespread application of orthobiologics in orthopedic surgery and sports medicine injuries. Our findings suggest that specific PRP preparations after a single freeze–thaw may be more advantageous for certain musculoskeletal applications based on the presence of MMP concentrations.

## Figures and Tables

**Table 1:** Key function and roles of matrix metalloproteinase (MMP) isoforms in musculoskeletal tissue remodeling.

MMP Isoform	Common Name	Role in Musculoskeletal Tissue Remodeling	References
*MMP-1*	*Interstitial Collagenase*	Collagenolytic MMP; degrades collagen I, II and (primarily) III in cartilage; increases protein turnover and collagen degradation in tendons and ligaments.	[24,35,36,37,38,39]
*MMP-2*	*Gelatinase* *-A*	Collagenolytic MMP; reabsorbs osteoclasts; widely expressed in healthy tissues.	[24,40,41]
*MMP-3*	*Stromelysin-1*	Degrades collagen and non-collagen, extracellular molecules (i.e., proteoglycans, fibronectin, elastin, etc.) to initiate tissue remodeling processes; upregulates in inflammatory settings.	[24,42,43,44,45]
*MMP-9*	*Gelatinase* *-B*	Removes attachment proteins and adhesion complexes from collagen networks; predominately active in angiogenic processes, especially in early stage of wound healing.	[24,38,39,43,44,45,46]
*MMP-10*	*Stromelysin-2*	Potentiates cartilage collagenolysis; known to promote cartilage and bone catabolism.	[47,48,49]
*MMP-12*	*Metalloelastase* *(Macrophage elastase)*	Primarily degrades elastin; reabsorbs osteoclasts and remodels fetal bone.	[41,50]

**Table 2:** Subject demographics. Values are reported as the median and range.

Age (years)	29 (23–60)
Gender	9 Male, 10 Female
BMI (kg/m^2^)	22 (19.7–27.9)

**Table 3:** Total platelet (PLT), white blood cell (WBC) and red blood cell (RBC) counts. Values are expressed as the median and range.

	PLTs (10^3^/μL)			WBCs (10^3^/μL)			RBCs (10^6^/μL)		
Time	Whole Blood	LP-I	LR-I	Whole Blood	LP-I	LR-I	Whole Blood	LP-I	LR-I
Fresh (baseline)	217 (157–292)	1218 (586–2261)	833 (564–1931)	5.4 (3.9–7.72)	2.4 (0.69–5.7)	12.5 (6.43 –26.3)	4.1 (3.7–4.9)	0.05 (0.01–1.23)	4.4 (0.03–7.4)

**Table 4:** Leukocyte differential. Values are expressed as the median and range.

	NEU (10^3^/μL)			LYM (10^3^/μL)			MONO (10^3^/μL)			EOS (10^3^/μL)		BASO (10^3^/μL)			
Time	Whole Blood	LP-I	LR-I	Whole Blood	LP-I	LR-I	Whole Blood	LP-I	LR-I	Whole Blood	LP-I	LR-I	Whole Blood	LP-I	LR-I
Fresh (baseline)	3.2 (2.5–4.41)	0.09 (0.01–3.17)	4.6 (0.03–17.5)	1.5 (0.90–2.3)	2.0 (0.49–10.2)	6.1 (3.6–11.7)	0.42 (0.25–0.62)	0.21 (0.09–1.92)	1.4 (0.60–2.3)	0.09 (0.03–0.44)	0.003 (0.00–0.39)	0.05 (0.01–1.1)	0.06 (0.03–0.13)	0.03 (0.00–0.32)	0.18 (0.01–0.36)

## Data Availability

The data presented in this study are available on request from the corresponding author.
